# Effects of chicken hemoglobin antimicrobial peptides on intestinal mucosal immunity under chronic heat stress and vaccination responses in broilers

**DOI:** 10.3389/fvets.2025.1574513

**Published:** 2025-06-09

**Authors:** Decheng Wang, Fengjiao Hu, Hui Liu, Ruiping She, Jijing Tian

**Affiliations:** Laboratory of Animal Pathology and Public Health, Key Laboratory of Zoonosis of Ministry of Agriculture, College of Veterinary Medicine, China Agricultural University, Beijing, China

**Keywords:** antimicrobial peptides, heat stress, intestinal mucosal immunity, chicken, vaccination

## Abstract

Heat stress (HS) is a major concern in poultry production worldwide due to its adverse effects on feed intake, weight gain, carcass weight, and metabolic conditions. Several strategies have been explored to ameliorate the negative effects of HS in broiler chickens, among which antimicrobial peptides (AMPs) represent a promising approach. Previously, we isolated chicken hemoglobin antimicrobial peptides (CHAP) and further demonstrated that CHAP has strong bactericidal activity. However, whether CHAP can improve growth performance and maintain intestinal mucosal immunity under chronic HS conditions remains unclear. In the present study, a total of 141 one-day-old broilers were divided into two groups. A total of 36 broilers were used to establish a chronic HS model to evaluate the effects of CHAP on intestinal mucosal immunity, and the remaining 105 birds were used to monitor the inductive effects of CHAP on two vaccines, including Newcastle disease virus (NDV) and avian influenza virus (AIV) vaccines, in broilers. As expected, HS-stimulated broiler chickens supplemented with CHAP showed a significant increase in villus height in the duodenum (*p* < 0.01), jejunum (*p* < 0.05), and ileum (*p* < 0.01) compared to those who did not receive CHAP under chronic HS conditions. The levels of alkaline phosphatase (AKP) and the number of secretory IgA (sIgA)-producing cells were markedly decreased in the chronic HS group (*p* < 0.01), whereas both significantly recovered after CHAP administration (*p* < 0.01). CHAP administration improved the birds' body weight and average daily gain (ADG), as well as the feed utilization rate, under HS conditions. Moreover, CHAP effectively mitigated HS-induced bursa injury by inhibiting excessive bursal apoptosis through the downregulation of caspase-3 and Bax, as well as the upregulation of Bcl-2 (*p* < 0.01). Interestingly, CHAP supplementation enhanced the antibody titer of both NDV and AIV in the broilers. Finally, CHAP administration enhanced the proliferation of splenic lymphocytes. In summary, our data demonstrate that CHAP not only maintains intestinal stability to improve growth performance but also inhibits excessive apoptosis in immune organs and upregulates vaccination effects.

## 1 Introduction

Global warming has increased the frequency of extreme heat events, with heat stress (HS) being one of the most significant environmental challenges affecting poultry production (1, 2). Some pioneering studies have demonstrated that the poultry industry has become highly sensitive to environmental temperature changes, and thereby, HS has become a critical factor restricting the healthy and sustainable development of the livestock and poultry sectors (3, 4). Existing studies have demonstrated that chronic HS not only breaks the homeostasis of bile acid metabolism and gut-associated microbiota in broilers (4) but also induces lung injury in broiler chickens by disrupting the integrity of the blood–air barrier (5). Two studies from different groups have clarified that chronic HS not only significantly impairs growth performance and intestinal mucosal barrier parameters in broilers (6) but also causes liver damage by triggering ER stress-induced excessive apoptosis (7). Moreover, some reports have demonstrated that HS considerably reduces feed intake and production (8, 9), as well as decreases sperm fertility, leading to infertility (10). Our previous study also found that chronic HS markedly reduced average daily gain (ADG) and feed efficiency while causing severe intestinal injury in broilers (11). It is now widely recognized that heat stress is one of the most detrimental environmental challenges affecting poultry production and welfare, causing huge economic losses in the poultry industry worldwide (12).

Antimicrobial peptides (AMPs), originally described as natural microbicides that are selectively cytotoxic to bacteria while exhibiting minimal cytotoxicity to mammalian host cells, are an important part of the innate immune system of different vertebrate organisms (13–16). As a class of small peptides, AMPs not only exhibit stronger inhibitory effects against bacteria, fungi, parasites, and viruses (14, 17–20) but also have stronger regulatory effects during various pathophysiological processes (16, 21–24). With the successive discovery of a variety of AMPs, an increasing number of studies have shown that AMPs are actively involved in rapid chemoattraction and/or modulate host cells to engage in immunoregulatory processes. One kind of antimicrobial peptide derived from the sacculus rotundus of rabbits has been shown to maintain intestinal mucosal immunity and protect chickens from infection with very virulent infectious bursal disease virus (25, 26). Swine gut-derived antimicrobial peptides have been shown to improve growth performance and intestinal absorption capacity (27), as well as maintain the integrity of the intestinal mucosal surface, in chickens (28). NKHs27, originally derived from *hyporthodus septemfasciatus*, significantly improves the respiratory burst ability of macrophages *in vitro* and limits pathogen dissemination *in vivo* (29). A synthetic amphipathic molecule designed to mimic the properties of cationic antimicrobial peptides (cAMPs) could combat SARS-CoV-2 infection by upregulating the expression of type I interferons or IL-6 via an immunomodulatory strategy (30).

Recently, biologically active peptides obtained from food byproducts or wastewater from poultry slaughtering facilities have garnered increasing attention. Gallego et al. isolated a bioactive peptide from Spanish dry-cured ham bones and found that it positively impacts cardiovascular health (31). Wu et al. (32) identified an antioxidant peptide from the skin of *Quasipaa spinosa* and found that frog skin-derived peptide derivatives have potential applications as functional foods. A recent report found that steam-exploded tilapia skin-derived peptides exhibit strong ACE inhibitory effects and antihypertension activity (33). Saffron petals, a byproduct of the procession of the crude drug saffron, were also shown to effectively alleviate colon tissue damage and prevent body weight loss in a DSS-induced colitis mouse model (34). Previously, our research team successively established methods for the isolation and extraction of antimicrobial peptides from the swine intestine and blood (11, 27, 28), as well as from the sacculus rotundus of rabbits (25). In addition to isolating biological peptides from swine byproducts, we also established an optimized method to purify chicken hemoglobin-derived antimicrobial peptides (CHAP). We found that CHAP not only can combat 19 bacterial strains, including nine multidrug-resistant bacterial strains, but also exhibits lower embryotoxicity and high stability across different temperatures (35). Although CHAP has demonstrated potent antimicrobial activity, little is known about its immunoregulatory effects on chickens under heat-stress conditions. The present study aimed to determine the effects of CHAP on intestinal mucosal immunity under chronic heat stress and vaccination responses in broiler chickens.

## 2 Materials and methods

### 2.1 Preparation of CHAP

CHAP was isolated as previously described by Hu et al. (35). In brief, fresh chicken blood was harvested from Beijing Huadu Broiler Corporation (Beijing, China). It was mixed with a tissue masher to break the blood cells and then subjected to enzymatic hydrolysis with papain. Then, 5% acetic acid was added to the extract and incubated overnight at 4°C. Subsequently, the supernatant was centrifuged at 8,000 × g for 30 min at 4°C, and pH was adjusted to 6.0 ± 0.5 to obtain the crude extract. After measuring the concentration, the crude CHAP was loaded onto a 10 × 300 mm Sephadex G-100 column, and the purified elution was collected as previously described (35). The final fractions of interest were collected and stored as lyophilized powders at −20°C.

### 2.2 Establishment of a chronic HS model and CHAP intervention in the broiler chickens

In artificial climate chambers, 36 one-day-old, specific-pathogen-free (SPF), Arbor Acre (AA) male broiler chickens with similar BW were selected from Beijing Merial Vital Laboratory Animal Technology Co., Ltd (Beijing, China; https://en.bi-vital.com/). All birds were randomly divided into three groups (12 chicks per group): the Control group, the chronic HS group, and the heat-stress with CHAP intervention (CHAP&HS) group. A total of four chickens were placed in each cage, with three replicates per group. All chickens were kept at 20°C for 3-day acclimation before the trial. Each bird in the CHAP&HS group was given 0.2 mg of CHAP dissolved in 1 ml of sterile 0.9% NaCl solution by gavage every morning. The chickens in the other groups received the same volume of sterile 0.9% NaCl solution. During the 10-d experiment, the birds in the HS and CHAP&HS groups were exposed to 35°C to simulate high-temperature conditions, as previously established by Hu et al. (11). The control group was continuously maintained at 20°C until the end of this experiment. All birds received the same diet and were provided feed and water *ad libitum*. Body weight and feed intake were measured and recorded, and ADG, the feed conversion ratio (FCR), and average daily feed intake (ADFI) were calculated subsequently. This study was reviewed and approved by the Institutional Animal Care and Use Committee of China Agricultural University.

### 2.3 Effects of CHAP on the broiler chickens vaccinated against NDV and AIV

A total of 105 one-day-old, SPF, AA broiler chickens were obtained from Beijing Merial Vital Laboratory Animal Technology Co., Ltd (Beijing, China). All 105 birds were randomly assigned to three groups (35 chickens per group): the normal control group (CON), the CHAP intramuscularly injected group [CHAP(I)], and the CHAP drinking-administrated group [CHAP(D)]. Then, all chickens were immunized with the Newcastle disease virus (NDV) vaccine via nasal drops (0.05 ml/chicken) on days 3 and 15, and the avian influenza virus (AIV) vaccine was administered by myocardial injection (0.1 ml/bird) on day 15. For the CHAP(I) group, CHAP was intramuscularly injected at a dose of 0.1 mg/kg body weight on days 1, 7, 14, 21, 28, 35, 42, and 49. For the CHAP(D) group, CHAP was given at 20 mg/L per chicken at the same time points. All animals were provided with the same feed and water *ad libitum*. This study was reviewed and approved by the Institutional Animal Care and Use Committee of China Agricultural University.

### 2.4 Sample collection and processing

For the chronic HS model, the weight of all chickens was recorded daily throughout the experimental period. At the final time point of CHAP administration, all birds were euthanized and the blood samples were collected immediately. Then, the spleen, thymus, and bursa were quickly removed and weighed. Subsequently, different intestinal parts were removed and collected as previously described. A 1 cm section of the duodenum was obtained from the proximal duodenum (~4 cm after the pylorus), a 1 cm section of the jejunum was collected from the proximal jejunum (~2 cm after the yolk stalk), and a 1 cm section of the ileum was cut from the middle part between Meckel's diverticulum and the ileocecal junction (27, 36). Finally, all harvested tissues were immediately fixed in a 2.5% (vol/vol) glutaraldehyde-polyoxymethylene solution as previously described (37, 38).

To assess the induction effects of CHAP on the vaccination responses to NDV and AIV in chickens, five chickens from each group were randomly selected on days 7, 14, 21, 28, 35, 42, and 49. Then, each chicken was euthanized, weighed, and recorded. Blood samples were collected first, followed by the removal of the chicken's organs, including the spleen, thymus, and the bursa of Fabricius, to calculate the organ index using the following formula: organ index = *W*_organ_/*W*_body_, where *W*_organ_ represents the weight of the organ (mg) and *W*_body_ represents the weight of the body (g) (39).

### 2.5 Histology analysis

The fixed tissues, including immune organs such as the bursa, spleen, thymus, and different intestinal parts of the duodenum, jejunum, and ileum, were dehydrated and embedded in paraffin using our standard laboratory protocol (38, 40). Then, 5 μm serial paraffin sections were cut and kept at 37°C to dry thoroughly. Subsequently, the 5 μm sections were dewaxed with a graded alcohol solution and stained continuously with hematoxylin and eosin.

### 2.6 Measurement of villus height, AKP, and GCs in the intestine

Five micrometer histological sections of the duodenum, jejunum, and ileum were stained with hematoxylin and eosin. Then, five views per section and five sections per chicken were randomly selected and measured using the Motic Med 6.0 CMIAS Image Analysis System (MOTIC CHINA GROUP CO., LTD., China). Finally, the villus height values were obtained, and the average was calculated to determine the mean villus height for each section as per our established protocols (11, 25, 27, 28, 38). Goblet cells (GCs) in the intestine were identified using the improved periodic acid-Schiff (PAS) staining method as previously described (11, 28, 38). The profiles of intestinal alkaline phosphatase (AKP) were measured using our optimized Gomori's calcium-cobalt amendment method (11, 27, 38). After staining, five fields per section and five sections per chicken were randomly captured using the Motic Med6.0 CMIAS Image Analysis System (MOTIC CHINAGROUP CO., LTD., China). The number of GCs and the area density of AKP were calculated for statistical analysis (11, 38).

### 2.7 TUNEL staining

The 5 μm serial sections of the bursa, spleen, and thymus were prepared as previously described (11). Then, apoptotic cells in the histological sections—including the bursa, spleen, and thymus—were identified using our improved TUNEL staining method (41, 42). Deparaffinized and rehydrated sections were stained with an *in-situ* apoptosis detection kit (DeadEnd™ Colorimetric TUNEL System, Promega) according to the manufacturer's instructions. After counterstaining with hematoxylin, apoptotic cells with brown and blue nuclei were identified by visualization in the TUNEL-stained sections. Finally, all mounted sections were reviewed blindly under an Olympus microscope. The TUNEL-positive (apoptotic) cells were counted, and five fields per section and five sections per chicken were randomly captured using the Motic Med6.0 CMIAS Image Analysis System (MOTIC CHINAGROUP CO., LTD., China).

### 2.8 Effect of CHAP on the proliferation of splenic lymphocytes

Fresh spleen samples from the NDV- and/or AIV-vaccinated chickens were harvested and immediately transferred to an aseptic cabinet for isolating splenic lymphocytes, as described in a previous report (43). In brief, splenic lymphocytes were prepared by homogenizing the spleen of all groups of chickens with a grinder and suspending the cells in a lysis buffer (0.15 M NH_4_Cl, pH 7.4) for 10 min to remove erythrocytes. Then, the mixture was centrifuged at 1,500 rpm for 30 min, and the supernatant was discarded. The obtained precipitate was gently washed twice with Hanks' solution (Solarbio, Beijing, China) and re-suspended in RPMI-1640 medium supplemented with 10% FBS. Cell viability was measured by trypan blue staining according to the established protocol (44). Then, the cells were seeded into 96-well plates (1 × 10^6^ cells/well) and treated with 20 μg/ml of Con A (20 μg/ml, Sigma Co., China) or LPS (20 μg/ml, Sigma Co., China) by adding 12 μl of the respective test solution. The control cultures received 12 μl of the culture medium without Con A or LPS. The cells were then cultured at 37°C in an atmosphere containing 5% CO_2_. After 24 h of incubation, cell viability was evaluated using a CCK-8 kit (Solarbio, Beijing, China). Finally, cell proliferation activity was measured using a commercialized WST-8 kit (Solarbio, Beijing, China) at 450 nm absorbance with a microplate reader. Cell proliferation activity was calculated as follows: Proliferation rate (%) = (P2 – P0)/(P1 – P0) × 100%, where P0 is the absorbance value of the medium containing Con A or LPS, P1 is the absorbance value of the control group, and P2 is the absorbance value of the CHAP-treated groups.

### 2.9 Immunological staining

The 5 μm histological sections of the duodenum, jejunum, and ileum were obtained and subjected to an avidin-biotin complex (ABC) immunohistochemistry examination for the detection of secretory IgA (sIgA, mouse-anti-chick IgA, Southern Technology Inc., Longwood, FL) and proliferating cell nuclear antigen (PCNA, rabbit anti-PCNA antibody, Beijing Biosynthesis Biotechnology CO., LTD., Beijing, China). Firstly, the histological sections were deparaffinized, rehydrated, and rinsed in PBS-T (0.01 mol/L PBS, pH 7.4). After being heated at 94°C in a citrate buffer solution (0.01 mol/L, pH 6.0) for antigen retrieval, the sections were allowed to cool naturally for 30 min, then immersed in 3% aqueous hydrogen peroxide at room temperature for 30 min for endogenous peroxidase ablation. The following steps were performed in a moist chamber. The sections were gently washed three times and incubated sequentially with the goat serum, the indicated primary antibody and its matched secondary antibody, and horseradish peroxidase-labeled avidin chain working fluid (Beijing Zhongshan Golden Bridge Biotechnology Co., Ltd., Beijing, China). Finally, five fields per section and five sections per chicken were randomly captured using the Motic Med6.0 CMIAS Image Analysis System (MOTIC CHINAGROUP CO., LTD., China). The number and area density of sIgA and PCNA were quantified for statistical analysis (28, 40).

### 2.10 Statistical analysis

The experimental data were expressed as mean ± standard deviation (SD), as indicated in the matched figure legends. For comparisons between two groups, unpaired, two-tailed Student's *t*-test was used. Cellular data were analyzed using One-way ANOVA or two-way ANOVA for multiple comparisons, while clinical data were analyzed using the Mann–Whitney *U*-test. A *p*-value <0.05 was considered statistically significant. Graphs are representative of at least three independent experiments.

## 3 Results

### 3.1 Effects of CHAP on daily gain and the feed conversion rate in the chickens under chronic heat stress

**Figure 1:**
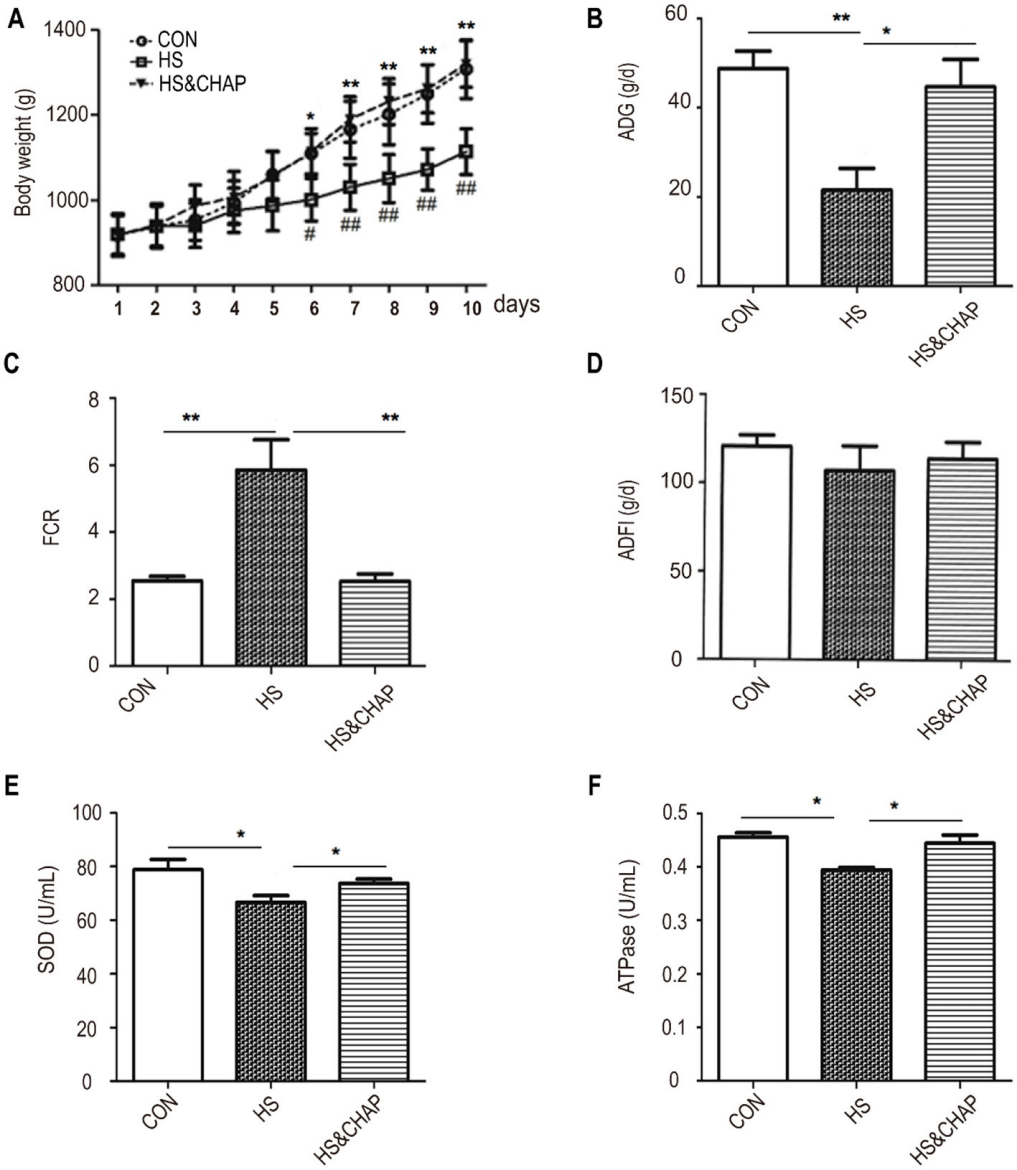
Effect of CHAP on the growth performance of the broiler chickens under chronic HS. **(A)** Dynamic changes in the body weight of the chickens under chronic HS conditions with or without CHAP supplementation. **(B–D)** Effects of CHAP on average daily gain (ADG), the feed conversion ratio (FCR), and average daily feed intake (ADFI) under chronic HS conditions, respectively. **(E, F)** Effects of CHAP on the expression levels of SOD and ATPase under chronic HS conditions, respectively. Data are presented as means ± SD. The * and ** indicate significant differences from the CON group at *p* < 0.05 and *p* < 0.01, respectively. The # and ## indicate significant differences between the HS and HS&CHAP groups at *p* < 0.05 and *p* < 0.01, respectively. CON, control group; HS, heat stress group; HS&CHAP, heat stress coupled with CHAP treated group.

Throughout the experimental period, the animals in the control group remained in good health, while chronic heat stress significantly reduced body weight and average daily gain and markedly increased the feed-to-gain ratio in the chickens. At the beginning of the study, there was no significant difference in body weight between the chronic HS group and the HS&CHAP group compared to the control group (*p* > 0.05; Figure 1A). After chronic HS was initiated in the broiler chickens, the body weight of the chickens in the HS group reduced significantly compared to the control group (*p* < 0.05). However, CHAP supplementation restored the body weight of the chickens, even under HS conditions (*p* < 0.05; Figure 1A). The animals in the chronic HS group exhibited lower average daily gain (ADG) and a higher feed conversion rate (FCR) compared to the control group (*p* < 0.05; *p* < 0.01). Unexpectedly, CHAP administration improved the ADG and reduced the FCR of the chickens under chronic HS conditions, showing a significant difference compared to the chronic HS group (*p* < 0.05; *p* < 0.01; Figures 1B, C). Surprisingly, there was no significant difference in the average daily feed intake (ADFI) of the chronic HS broilers compared to the control group or CHAP-intervened groups (Figure 1D). Moreover, superoxide dismutase (SOD) and ATPase levels were significantly reduced in the HS group compared to the control group, while CHAP intervention upregulated the levels of SOD and ATPase in the broilers, even under HS conditions (Figures 1E, F). The above results indicate that the chronic HS model had a significant inhibitory effect on the production performance of the chickens.

### 3.2 Effects of CHAP on intestinal morphology under chronic conditions

**Figure 2:**
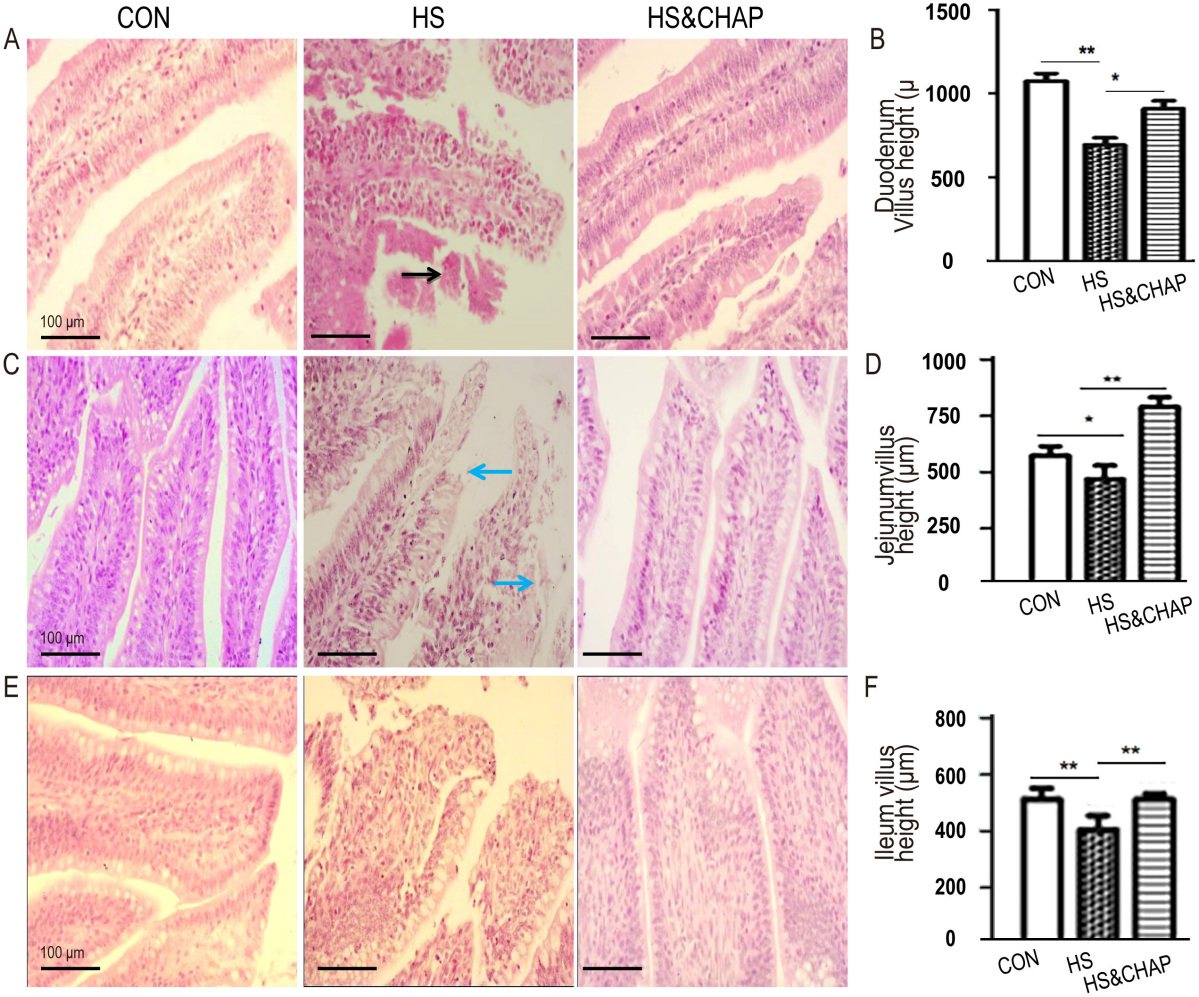
Effects of CHAP on villus height in different parts of the intestine. **(A, B)** Effects of CHAP on villus height in the duodenum under chronic HS stimulation. In the control group, the intestinal villus epithelium was arranged neatly and the villi were intact and longer. Under chronic HS conditions, the duodenal villi appeared disordered and shortened. Some villi were entangled in mucus, and hemorrhage occurred in the lamina propria and (or) submucosa (black arrow indicated). However, supplementation with CHAP in drinking water increased villus height in the duodenum. **(C, D)** CHAP administration effectively improved villus height in the jejunum, even under chronic HS conditions. **(E, F)** CHAP treatment alleviated the damage caused by chronic HS in the ileum. Data are presented as means ± SD. The * and ** indicate significant differences at *p* < 0.05 and *p* < 0.01, respectively. CON, control group; HS, heat stress group; HS&CHAP, heat stress coupled with CHAP treated group.

Compared to the control group, the chickens under chronic HS had reduced villus height in the duodenum (*p* < 0.01; Figures 2A, B), jejunum (*p* < 0.05; Figures 2C, D), and ileum (*p* < 0.01; Figures 2E, F). Moreover, histopathological examination showed that the villus epithelium and the lamina propria of the duodenum and jejunum were edematous, and some villi were shed under chronic heat stress (Figures 2A, C). As expected, the chickens that were given CHAP showed an increase in villus height and recovered intestinal structure in the duodenum (*p* < 0.01; Figures 2A, B), jejunum (*p* < 0.01; Figures 2C, D) and ileum (*p* < 0.01; Figures 2E, F) compared to the chronic HS group.

### 3.3 Effects of CHAP on the expression profile of AKP and sIgA under chronic conditions

**Figure 3:**
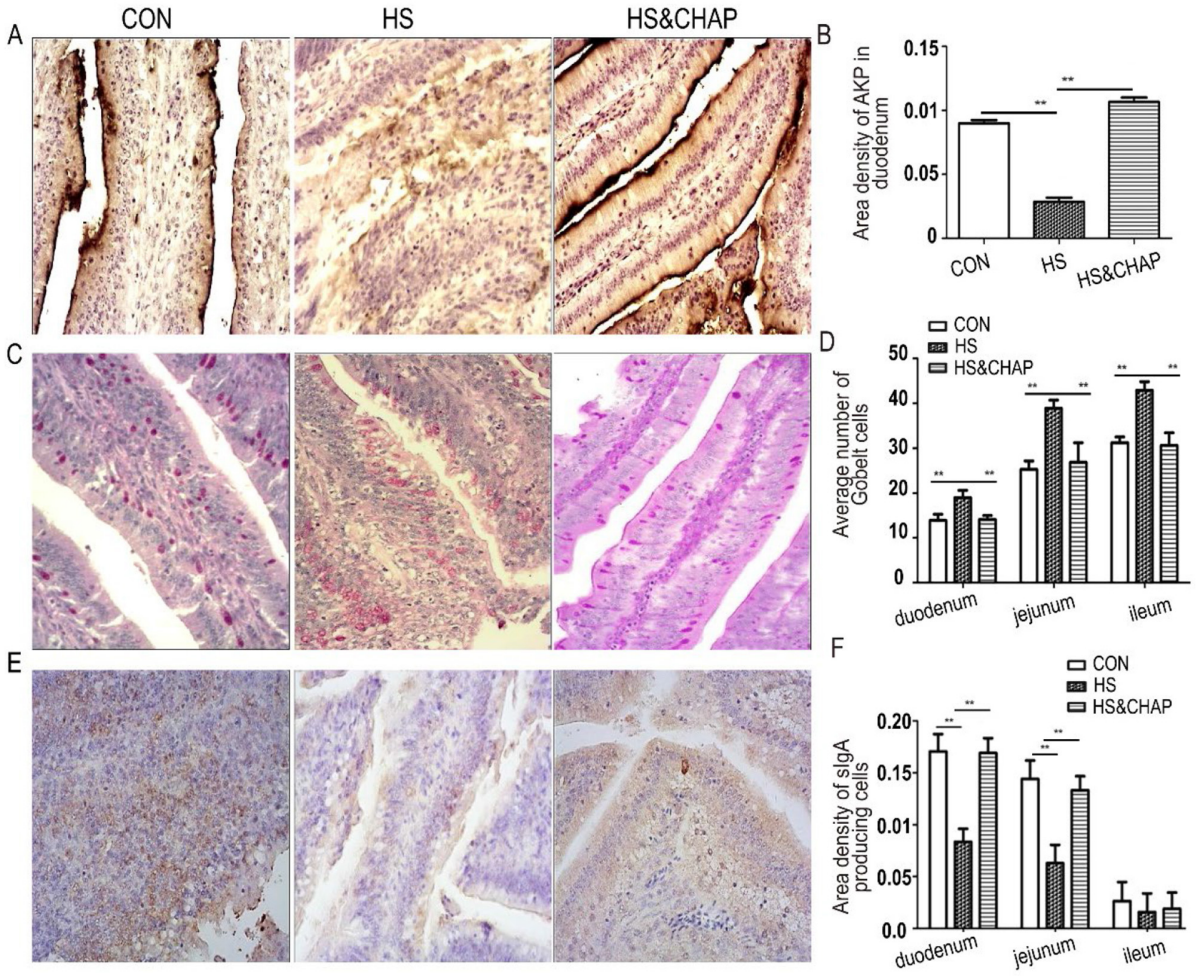
Effects of CHAP on intestinal AKP, GCs, and sIgA. **(A, B)** Gomori's calcium-cobalt staining found reduced expression of AKP in the HS group; however, CHAP administration significantly restored AKP production in the chickens, even under chronic HS conditions. **(C, D)** Improved PAS staining showed an increased density of GCs in the HS group; however, most of the GCs appeared incomplete, with their interior materials partially released. **(E, F)** Immunohistochemistry staining of sIgA and its quantitation. The ** indicate a significant differences at *p* < 0.01. CON, control group; HS, heat stress group; HS&CHAP, heat stress coupled with CHAP treated group.

Intestinal AKP is a resident brush-border enzyme that is crucial for the absorption and transport of nutrients and the maintenance of intestinal homeostasis (45–47). Considering that chronic heat stress reduced intestinal villus height, the AKP levels were measured. We found that AKP was highly expressed in the duodenum, but chronic heat stress significantly reduced its expression (*p* < 0.01; Figures 3A, B). Unexpectedly, AKP expression was restored in the duodenum of the chickens that received CHAP at 20 mg/L in the drinking water, even under chronic heat stress (*p* < 0.01; Figures 3A, B). Using established PAS staining (11, 27, 38), we found that the GCs displayed a typical goblet shape within the columnar cells. The number of GCs was significantly increased in the HS group, and there was a significant difference compared to the control group or HS&CHAP group (*p* < 0.01; Figures 3C, D). In each fragment of the intestinal samples, the sIgA-secreting cells were mainly distributed within the mucosa epithelium that featured a nucleus surrounded by a ring of yellow-brown cytoplasm (Figure 3E). In both the duodenum and jejunum, the area density of sIgA-producing cells in the chronic HS group was significantly reduced compared to the control group (*p* < 0.01; Figure 3F), while CHAP administration effectively recovered sIgA expression in the chickens under HS stimulation (*p* < 0.01; Figure 3F).

### 3.4 Effects of CHAP on chronic HS-induced apoptosis in the chickens' immune organs

**Figure 4:**
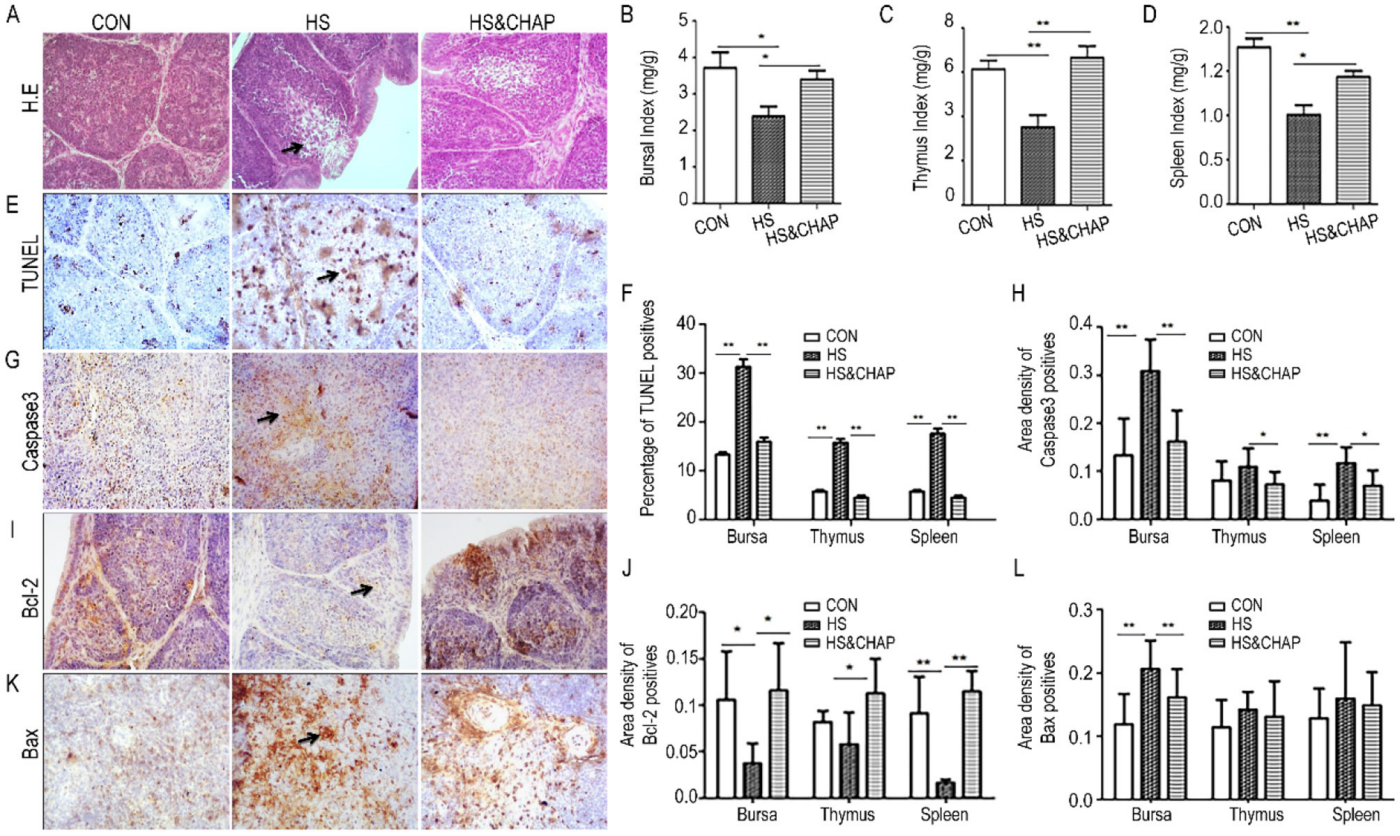
Effects of CHAP on apoptosis in the immune organs under chronic HS conditions. **(A)** H.E. staining found that the bursa of Fabricius was swollen and that a large number of necrotic lymphoid follicles and immune cells were present in the chronic HS group. **(B–D)** Quantitation demonstrated that chronic HS reduced the organ index of major immune organs, including the bursa, spleen, and thymus. **(E, F)** TUNEL staining showed excessive apoptotic positive cells in the chronic HS-treated group, while CHAP administration reduced apoptosis. **(G, H)** Immunohistochemistry staining demonstrated an increased expression of Caspase-3 in the bursal tissues of the HS group. **(I, J)** Bax, a pro-apoptotic protein, was significantly upregulated in the bursa under chronic HS conditions; however, it was dramatically decreased following CHAP treatment. **(K, L)** Bcl-2, an anti-apoptotic molecule, was downregulated in the HS group, but it was recovered after CHAP administration. The * and ** indicate significant differences at *p* < 0.05 and *p* < 0.01, respectively. CON, control group; HS, heat stress group; HS&CHAP, heat stress coupled with CHAP treated group.

Histopathological examination showed that numerous follicular lymphocytes were absent in the bursa of Fabricius of the HS group (Figure 4A), statistically indicating that the organ index—including the bursa, spleen, and thymus—was significantly reduced in the HS group (Figures 4B–D) compared to the control and HS&CHAP groups (*p* < 0.05; *p* < 0.01). Administering CHAP to the chickens effectively restored those organs under chronic HS stimulation (Figures 4B–D). TUNEL staining revealed a large amount of brown signal distributed in the bursa of the animals under chronic HS conditions; however, fewer TUNEL-positive cells were observed in the control and HS&CHAP groups (Figure 4E). Statistical quantitation demonstrated that there was a significant difference in the HS group compared to the control and HS&CHAP groups (Figure 4F). Some critical pro-apoptotic or anti-apoptotic factors such as Caspase-3, Bcl2, and Bax were determined using immunohistochemistry staining. Consistent with the excessive TUNEL-positive cells in the HS group, Caspase-3 and Bax, both pro-apoptotic factors, were highly expressed in the bursa and showed a significant difference compared to the control group (*p* < 0.05; Figures 4G–J). However, CHAP intervention significantly reduced the expression of Caspase-3 and Bax, even under chronic HS conditions (*p* < 0.05; Figures 4G–J). Bcl-2, a well-known anti-apoptotic molecule, was highly expressed in both the control and HS&CHAP groups, whereas it was rarely detected in the bursal tissues of the chickens under chronic HS conditions (*p* < 0.05; Figures 4K, L).

### 3.5 Effects of CHAP on the immune response induced by NDV and AIV vaccination

**Figure 5:**
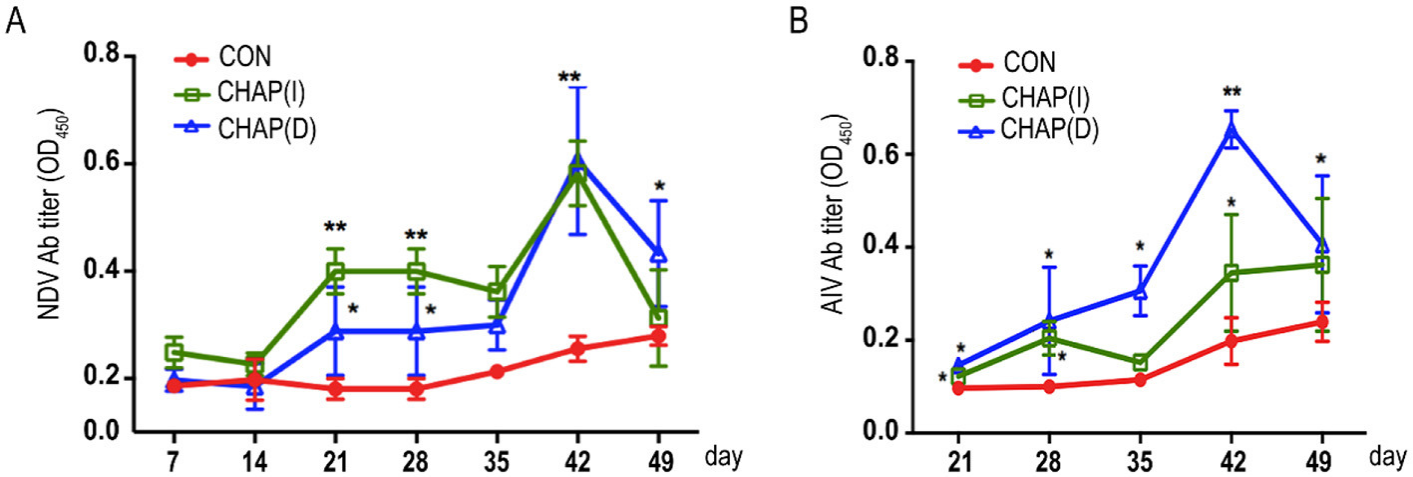
Effects of CHAP on the induction of vaccination responses to NDV and AIV. **(A)** The Newcastle disease virus (NDV) Ab titers were determined using a commercial ELISA kit (Finde, Shenzhen, China). The results showed that the NDV Ab titers in both CHAP-treated groups were higher compared to the control group (*p* < 0.05, *p* < 0.01). **(B)** CHAP administration significantly upregulated the concentration of the AIV antibody in the broiler chickens compared to the control group (*p* < 0.05, *p* < 0.01). Injected intramuscularly = CHAP(I), drinking water = CHAP(D). ^*^p < 0.05, ^**^p < 0.01.

The NDV antibody or AIV antibody was determined using the indicated commercial ELISA kit (Finde, Shenzhen, China) according to the manufacturer's instructions. As shown in Figure 5A, CHAP injected intramuscularly [CHAP(I)] significantly upregulated antibody titers in the broilers immunized with the NDV vaccine starting from day 21, compared to the chickens without CHAP (*p* < 0.05; *p* < 0.01). In contrast, the CHAP drinking group showed a significant difference on day 42 compared to the control (*p* < 0.05). For AIV vaccination shown in Figure 5B, both CHAP(D) and CHAP(I) enhanced induction in the broilers starting from day 21 compared to the matched control group (*p* < 0.05; *p* < 0.01).

### 3.6 Effects of CHAP on splenic lymphocytes proliferation

**Figure 6:**
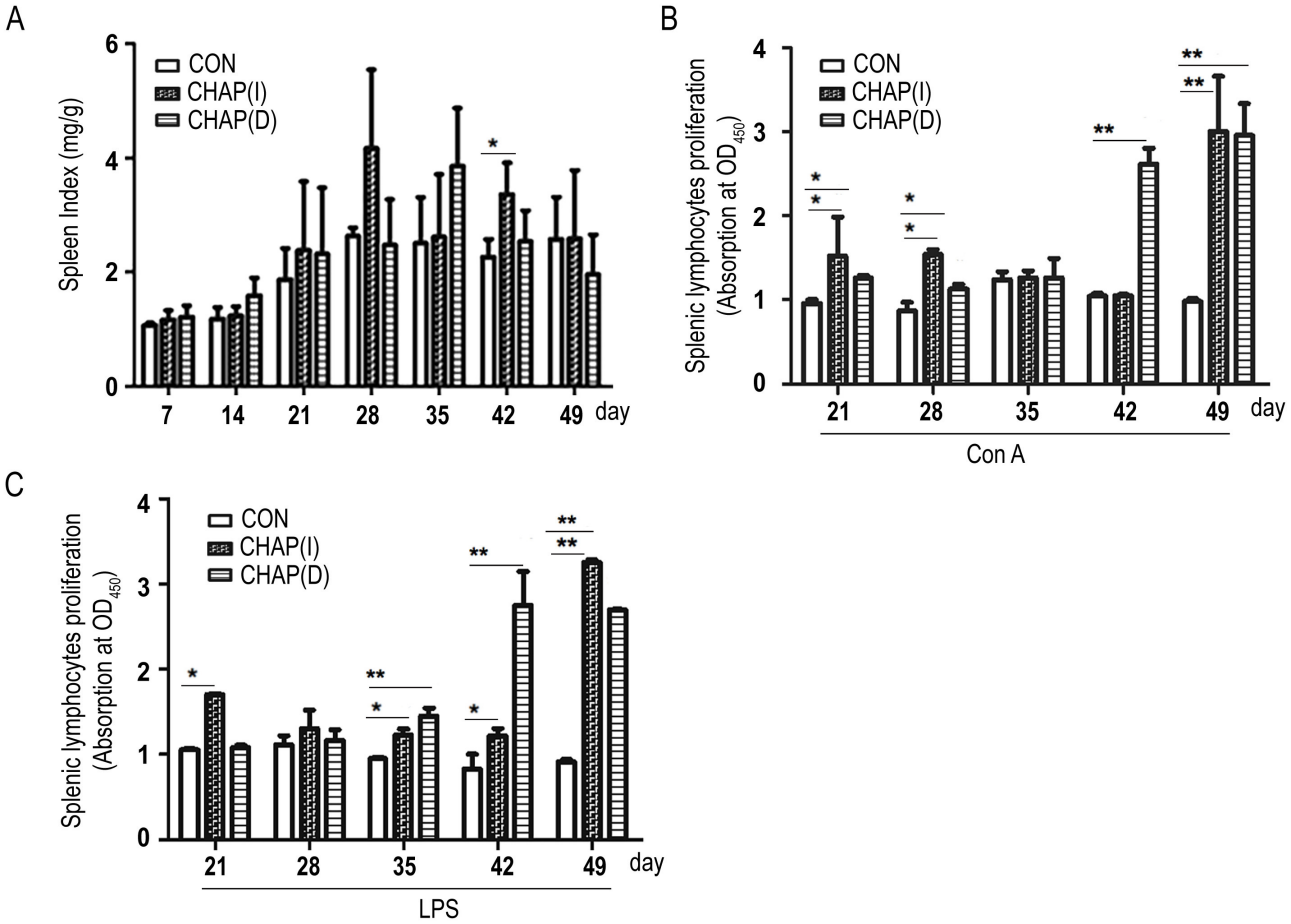
Effects of CHAP on the proliferation of splenic lymphocytes. **(A)** CHAP treatment improved the spleen index and showed a significant difference compared to the control group (*p* < 0.05). **(B, C)** CHAP-treated chicken splenic lymphocytes exhibited a higher proliferation rate compared to the control group under Con A or LPS stimulation (^*^p < 0.05, ^**^p < 0.01).

CHAP supplementation improved the chicken's spleen index compared to the control group (*p* < 0.05; Figure 6A). To further investigate the effect of CHAP on the proliferation of spleen lymphocytes, splenic lymphocytes were isolated from the spleen of all groups of chickens and treated with ConA or LPS to establish an inflammatory state. As shown in Figure 6B, after treatment with ConA, the splenic lymphocyte proliferation rate was increased in the CHAP-treated chickens starting from day 21, with a significant difference compared to the birds without CHAP supplementation (*p* < 0.05; *p* < 0.01). For LPS induction, the splenic lymphocyte proliferation rate was significantly increased on days 21, 35, 42, and 49 in the CHAP-treated group compared to the groups without CHAP (Figure 6C; *p* < 0.05; *p* < 0.01). These results indicate that CHAP could promote the proliferation of spleen lymphocytes in broiler chickens.

## 4 Discussion

Rising global temperatures are leading to an increased frequency of extreme heat events, with heat stress being the most significant (1, 2, 10, 48, 49, 76). With the growing number of reports on the hazards of heat stress, its potentially significant impact on the livestock and poultry industry has attracted considerable attention. Numerous studies have found that chronic heat stress not only modifies physiological parameters and meat quality but also induces liver injury in broiler chickens (50, 51). Heat stress also decreases productivity and poses a significant negative impact on pig production (52). Moreover, chronic HS exposure not only mediates hepatic lipid deposition but also induces renal fibrosis and mitochondrial damage in growing laying hens (53, 54). Our previous study demonstrated that chronic HS negatively affected intestinal mucosal functions and reduced the growth performance of broiler chickens (55). To reduce the losses caused by heat stress in the poultry industry, numerous exploratory studies have been conducted. Yin et al. (54) found that dietary *vitamin C* supplementation effectively alleviated hepatic lipid deposition in broiler chickens under chronic HS exposure. A trial study implied that dietary supplementation with dimethyl itaconate could alleviate oxidative stress and inflammation in broilers under chronic heat stress (56). Similarly, a recent study demonstrated that resveratrol could improve liver antioxidant function and promote growth performance in broilers exposed to HS conditions (57). Our previous study found that an isolated antimicrobial peptide from the swine gut effectively maintained growth performance in broiler chickens even under chronic HS (11).

In the present study, we investigated the effects of CHAP on growth performance and intestinal immune parameters in broiler chickens under HS conditions. Consistent with previous findings, chronic HS exposure not only significantly reduced body weight, average daily gain, and the feed conversion ratio in the broiler chickens but also decreased villus height in the duodenum, jejunum, and ileum. Our findings are in line with published reports indicating that chronic HS could impair gastrointestinal histology and intestinal mucosal barrier function in broilers (6, 9). Furthermore, histological analysis demonstrated that CHAP supplementation, administered via drinking water, increased villus height and improved growth performance under chronic heat stress conditions. This finding is in agreement with our previous reports, which showed that an isolated antimicrobial peptide from the swine gut had similar effects on broiler chickens (11, 27).

Intestinal AKP, an endogenous protein secreted by the microvilli of the intestinal epithelium, plays an essential role in intestinal homeostasis via balanced intestinal inflammation and intestinal permeability (47, 58, 59). Reduced AKP expression has been observed not only in chickens infected with the very virulent infectious bursal disease virus (38) but also in various parts of the intestine in broiler chickens under chronic HS stimulation (11). In the current study, intestinal AKP expression was significantly decreased in the chronic HS-treated group. However, the administration of CHAP remarkably improved the intestinal AKP profile in the harmful HS environment, implying that CHAP could maintain normal intestinal morphology and ensure effective nutrient absorption for growth performance under HS conditions. In other words, the present study, which found that CHAP upregulated AKP expression, is consistent with our previous research, where a swine gut-derived antimicrobial peptide effectively enhanced AKP activity to potentiate digest function and absorption (27).

Our previous study found that sIgA expression in the duodenum, jejunum, and ileum was significantly reduced in SPF chickens infected with the very virulent infectious bursal disease virus (38). Goblet cells (GCs) and secretory IgA (sIgA) are major components and multifaceted players of the intestinal immune barrier in maintaining intestinal homeostasis (60–63, 77). GCs reside in the epithelium and serve as the primary site for synthesizing and secreting mucus, which protects the intestinal mucosal layer from dehydration, damage, and various stimuli (60, 61). In the present study, HS stimulation significantly upregulated the number of GCs, suggesting that HS-triggered changes in GCs led to an increase in their number, allowing for the secretion of large amounts of substances to adapt to gut activity under HS conditions. This finding is consistent with our previous reports showing that chronic HS increased the number of GCs (11), but it is in disagreement with the finding that decreased GC numbers were observed in Cobb broilers under HS conditions (64). This suggests that there are some intrinsic differences in the morphology and function of GCs among different kinds of chickens.

Although some previous studies have demonstrated that HS impairs the development of immune organs, including the bursa, spleen, and thymus, in broiler chickens (65, 66, 78, 79), the underlying mechanisms contributing to these processes are complicated and not yet fully understood. In the present study, our data demonstrated that the immune organ indexes of the bursa of Fabricius and thymus were significantly decreased following chronic HS stimulation, which is consistent with some published reports suggesting that HS impairs immune organ indexes to disrupt immune system function (66). Moreover, some reports suggested that HS induced higher expression levels of pro-apoptotic genes, including Caspase-3 and Bax, in broiler livers. However, these genes were downregulated after dietary supplementation with dimethyl itaconate (56). Consistent with established publications, we found that HS stimulation caused considerable apoptosis in the bursa of the chickens, in which TUNEL-positive cells, Caspase-3, and Bax were significantly upregulated. As important pro-apoptotic effectors, the highly expressed Caspase-3 and Bax proteins promote the occurrence of apoptosis (67). Unexpectedly, CHAP administration in the HS-stimulated broilers markedly reduced Caspase-3 and Bax in the bursa, while it promoted the expression of Bcl-2. In the present study, CHAP supplementation upregulated Bcl-2 to prevent excessive apoptosis in the bursa of the broilers under HS conditions. Meanwhile, Caspase-3 and Bax were markedly reduced after CHAP administration, which is consistent with a recently published report suggesting that rabbit sacculus rotundus-derived antimicrobial peptides could inhibit excessive apoptosis of bursal lymphocytes to reduce follicle depletion and destruction mediated by vvIBDV-infection (26). Collectively, CHAP could inhibit excessive apoptosis to mitigate immune organ injury by downregulating the expression of Caspase-3 and Bax, as well as enhancing Bcl-2 expression, in broiler chickens under HS stimulation. CHAP, a kind of antimicrobial peptide derived from chicken hemoglobin, was first isolated by our research team and has shown potent and rapid antimicrobial activity, along with lower toxicity and high stability under different temperature conditions (35). A recent report found that daily supplementation with melittin in ducks could alleviate HS-induced immune organ damage and improve growth performance (68). In addition, dried plum supplementation was reported to decrease the negative effects of HS in broiler chickens by upregulating heat-shock-associated genes and nutrient transporters (69). Cathelicidin LL-37, a widely studied antimicrobial 37-mer peptide with various ascribed functions, was reported to be able to prevent HS-induced intestinal damage and heat-related illnesses in rats (70). Our data showed that in the chronic HS model, CHAP was capable of maintaining normal intestinal morphology, particularly protecting villus height and the AKP expression profile against heat stress-induced injury, which is important for nutrient absorption and the growth performance of chickens. Collectively, the present data demonstrate that those bioactive peptides have positive effects on growth performance, intestinal mucosal immunity, and its functions, which are consistent with our previous findings (11, 25, 27, 28, 35, 80–84). Therefore, these beneficial effects of CHAP may lead to more successful management of systemic inflammation and multiple organ injury in broiler chickens under HS conditions.

Bioactive peptides were separated from chicken byproduct hydrolysates in slaughterhouses and exhibited biological activity, offering several health benefits (71, 72). Keratinase is obtained by effectively utilizing various poultry wastes, including feathers, and provides practical insights and novel approaches for recycling and degrading biomass (73). Moreover, some biological peptides isolated from fishery byproducts, such as frames, trimmings, and viscera, showed antioxidant activity (74). Considering the large amount of poultry blood produced each year, our research team previously isolated a bioactive peptide from chicken blood and established a practical protocol (35). The present data further demonstrate that CHAP has positive effects on growth performance, and the action mechanism of CHAP is further revealed.

## 5 Conclusion

In conclusion, this report provides a more in-depth study of our previous work on CHAP. CHAP administration improves growth performance, intestinal mucosal immunity, and vaccination responses in broilers under chronic HS. It protects against HS-induced immune organ damage by regulating apoptosis-related proteins and enhances nutrient absorption by maintaining intestinal integrity. These findings highlight the potential of CHAP as a therapeutic agent for improving poultry production under heat stress conditions.

## 6 Limitations of this study

Although the present study provides valuable insights into the effects of CHAP on growth performance, intestinal mucosal immunity, and immune organs, some limitations remain and should be clarified in future studies. For instance, although CHAP modulated apoptosis-associated genes, including Caspase-3, Bax, and Bcl-2, under HS stimulation, more evidence is needed to determine whether the interaction between them is direct or indirect. Although CHAP showed protective effects in broiler chickens under HS conditions, its potential adverse effects should be considered in future studies. According to a published report, Cathelicidin LL-37 acted as a double-edged sword, promoting wound healing but with some derivatives exhibiting higher hemolysis and cytotoxic effects during this process (75). Therefore, further evidence is required for the assessment of CHAP-derived derivatives under HS stimulation. Finally, although CHAP functionally mitigated immune organ damage and upregulated the antibody titers induced by NDV and AIV vaccination, these findings are based on laboratory data and require additional validation. More controlled experiments and validation data, especially from commercial chicken farms, are indeed needed.

## Data Availability

The original contributions presented in the study are included in the article/supplementary material, further inquiries can be directed to the corresponding author.
